# Determinants of implementation of maternal health guidelines in Kosovo: mixed methods study

**DOI:** 10.1186/1748-5908-8-108

**Published:** 2013-09-09

**Authors:** Sharon E Straus, Julia E Moore, Sami Uka, Christine Marquez, A Metin Gülmezoglu

**Affiliations:** 1 Li Ka Shing Knowledge Institute, St. Michael’s Hospital, 30 Bond Street, Toronto, ON M5B 1W8, Canada; 2 Institute of Public Health, University Clinical Center, St. ‘Nëna Terezë,’ Rrethi i Spitalit pn, Pristina, Kosovo; 3 Department of Reproductive Health and Research, World Health Organization, 1211, Geneva 27, Switzerland

**Keywords:** Guideline implementation, Determinants of evidence uptake

## Abstract

**Background:**

One of the challenges to implementing clinical practice guidelines is the need to adapt guidelines to the local context and identify barriers to their uptake. Several models of framework are available to consider for use in guideline adaptation.

**Methods:**

We completed a multiphase study to explore the implementation of maternal health guidelines in Kosovo, focusing on determinants of uptake and methods to contextualize for local use. The study involved a survey, individual interviews, focus groups, and a consensus meeting with relevant stakeholders, including clinicians (obstetricians, midwives), managers, researchers, and policy makers from the national Ministry of Health and the World Health Organization office in Pristina, Kosovo.

**Results:**

Participants identified several important barriers to implementation. First, lack of communication between clinicians and ministry representatives was seen as leading to duplication of effort in creating or adapting guidelines, as well as substantial mistrust between clinicians and policy makers. Second, there was a lack of communication across clinical groups that provide obstetric care and a lack of integration across the entire healthcare system, including rural and urban centers. This fragmentation was thought to have directly resulted from the war in 1998 – 1999. Third, the conflict substantially and adversely affected the healthcare infrastructure in Kosovo, which has resulted in an inability to monitor quality of care across the country. Furthermore, the impact on infrastructure has affected the ability to access required medications consistently and to smoothly transfer patients from rural to urban centers. Another issue raised during this project was the appropriateness of including guideline recommendations perceived to be ‘aspirational’.

**Conclusions:**

Implementing clinical practice guidelines in low- and middle-income countries (LMICs) requires consideration of several specific barriers. Particularly pertinent to this study were the effects of recent conflict and the resulting fragmentation of healthcare and communication strategies among relevant stakeholders. However, as Kosovo rebuilds and invests in infrastructure after the conflict, there is a tremendous opportunity to create comprehensive, thoughtful strategies to monitor and improve quality of care. To avoid duplication of effort, it may be beneficial for LMICs to share information on assessing barriers as well as on guideline implementation strategies.

## Background

Around the world, health systems fail to optimally use evidence, which results in inefficiencies and reduced quantity and quality of life [1-6]. Recognition of this situation has created interest in knowledge translation (KT) and the need to enhance capacity in KT to meet the demand worldwide.

One of the most challenging KT problems is to reduce high rates of maternal and perinatal mortality and morbidity, especially in low- and middle-income countries (LMICs). At the global level, the maternal mortality rate has decreased at an average of less than 1% annually between 1990 and 2005, far below the 5.5% annual decline that would be necessary to achieve United Nations (UN) Millennium Development Goal 5, which focuses on improving maternal health. The Special Program of Research, Development, and Research Training in Human Reproduction (HRP) is a joint effort of the United Nations Development Program, the United Nations Population Fund, the United Nations Children’s Fund, the World Health Organization (WHO) and the World Bank. The HRP and its partners have developed a collaboration to tackle this challenge, adopting the ‘knowledge to action’ framework [7] to outline their approach. Known as the GREAT Project (for **G**uideline development, **R**esearch priorities, **E**vidence synthesis, **A**pplicability of evidence, and **T**ransfer of knowledge), this initiative includes integration of WHO recommendations into national guideline adaptation processes to expand access to effective practices at healthcare facilities and thus to improve maternal and perinatal health. The key goal of this initiative is to use evidence-based clinical practice guidelines to transfer high-quality evidence about maternal and perinatal health to stakeholders across the healthcare system. A rigorous process for guideline development, including representation from relevant stakeholders, has been used, including use of the GRADE (Grades of Recommendation Assessment, Development and Evaluation) approach for appraising the quality of evidence and determining the strength of recommendations [8]. To date, guidelines have been developed on prevention and management of postpartum hemorrhage, labor induction, hypertension in pregnancy, and the use of lay health workers for improving maternal and perinatal care [9].

Rather than focusing on the implementation of a single practice guideline, this initiative is tackling the development and evaluation of a broader implementation strategy that will include a multi-component intervention targeting the reduction of maternal and perinatal deaths. The strategy will span community- and hospital-based care and will consider improvements in access to care and in the referral process. The goal is to test this framework in a specified setting and to build local KT capacity to facilitate future initiatives.

The pilot site for this program is Kosovo. In that country, more than 95% of mothers give birth in a healthcare facility, but maternal morbidity and mortality remain greater than in many other European countries. The perinatal mortality rate in 2009 was 19.3%, and maternal mortality increased from 28.4 per 100,000 in 2005 to 43.3 per 100,000 in 2009 [10]. The most common causes of maternal mortality were infection and postpartum hemorrhage, both preventable with appropriate care.

The UN agencies in Kosovo are collaborating with local health authorities to achieve Millennium Development Goals 4 (reduce child mortality) and 5 (improve maternal health) [11]. It is expected that this process will lead to greater awareness and enhanced demand for reproductive healthcare services, improved access to high-quality reproductive healthcare and child health services, reduction and prevention of morbidity and mortality from major diseases among women and children, and increased knowledge and improved clinical skills of healthcare providers, which in turn will lead to strengthened caring practices and service delivery within individual facilities. Although the HRP has achieved success on many fronts, the need to optimize quality of care for mothers and infants remains an ongoing challenge.

One of the key challenges in implementation work is the need to adapt guidelines to the local context. By adapt we mean the process used to modify the guideline; it requires consideration of the local context. Work by the ADAPTE Collaboration [12] and the Canadian Guideline Adaptation Study Group (CAN-IMPLEMENT resource) [13] has provided frameworks for such adaptation; CAN-IMPLEMENT has been used to adapt various cancer guidelines [14,15], but this framework has not been validated. We undertook the current project to explore how these frameworks might be used in the setting of guideline implementation in an LMIC. We focused specifically on optimizing maternal health and started with an attempt to understand how guidelines should be contextualized through an understanding of barriers and facilitators to their implementation.

## Methods

To explore implementation of guidelines, including determinants of uptake and methods of contextualization, we performed a multiphase project encompassing a survey, individual interviews, focus groups, and an in-person consensus meeting. The guidelines selected for this project were the WHO guidelines on prevention and management of postpartum hemorrhage [16,17]. We used the CAN-IMPLEMENT model [13] to frame the approach to guideline adaptation and the knowledge to action framework [7] to model the implementation strategy.

### Setting

This study was focused in Kosovo and was conducted within the GREAT initiative framework, and represents a partnership with the Li Ka Shing Knowledge Institute of St. Michael’s Hospital in Toronto, the WHO headquarters in Geneva and its Kosovo office.

### Design

#### Phase one: survey

Using the approach suggested by CAN-IMPLEMENT, we developed a survey, including questions about respondents’ awareness of guidelines on the prevention and management of postpartum hemorrhage and the induction of labor created by various organizations (including the WHO), and asked respondents to rate the relevance and importance of these guidelines on a seven-point Likert scale [Additional file 1]. Along with the survey (translated into Albanian), respondents were given a copy of the WHO guidelines (also translated into Albanian) and links to the other guidelines. This step was done as part of the CAN-IMPLEMENT process, which suggests awareness of relevant guidelines be assessed and used to identify ‘buy in’ for implementation of a guideline. Before distribution to respondents, a draft of the survey was sent to three people (clinicians who were not involved with this study) to assess face validity [18].

The survey was sent to various stakeholders, including obstetricians and midwives, using two methods: direct email to 50 people identified through the local WHO office in Pristina, the capital of Kosovo (with reminders at two and four weeks, using Dillman’s methods to optimize the response rate [19]); and through the websites of various organizations, such as the Kosovo Ministry of Health, the Center for Family Medicine Development, Students of Medicine (University of Pristina) and the Center for Continuing Nursing Education). Participants were offered the option of completing the survey online or on paper. Consent was implied by completion of the survey, and respondents who completed the survey were offered a gift card. We calculated descriptive statistics for each item.

#### Phase two: interviews

We completed a series of telephone interviews with local stakeholders in Kosovo to develop key messages from the WHO guidelines and to understand local barriers and facilitators to their implementation. We used the results of the survey to inform development of the interview guide [Additional file 1]. Once informed consent was obtained, each participant was sent a package containing the WHO guidelines and the outline for the interview, with a request to consider each guideline, to nominate key messages and to describe barriers and facilitators to its implementation. These materials were available in English and Albanian.

Healthcare managers, healthcare professionals, lay healthcare workers, and policy makers were purposively sampled for inclusion. Snowball sampling was used to identify additional participants. Participants were initially identified through the WHO Liaison Office for Kosovo (Dr. Sami Uka), the Ministry of Health and the Kosovo Obstetrics and Gynecology Association (Prof. Dr. Shefqet Lulaj).

The interviews were conducted in English by an experienced interviewer. Each interview was digitally recorded, the recordings were transcribed, and the recording for each interviewee was assigned a unique identifier. Content analysis of the transcripts began after the first interview was completed [20,21]. Two investigators (CM, SES) independently read each transcript, using a constant comparative approach to identify themes [20]; this process was informed by the review of barriers to guideline implementation by Cabana et al. [22] The investigators then used these themes to develop codes. The credibility of the categories was determined by the frequency and consistency with which they were indicated by participants in their respective interviews. Themes were discussed iteratively within the team. The intention was to continue sampling until no new themes were identified; however, because of challenges in conducting interviews in English with stakeholders who spoke primarily Albanian and because of technical challenges with the telephones, the interviews were discontinued and plans were made to complete in-person focus groups with a translator.

#### Phase three: focus groups

We completed two 90-minute, in-person focus groups in Pristina in October 2012. The goal was to explore the barriers and facilitators to implementing WHO guidelines for postpartum hemorrhage. Once informed consent was obtained, participants were sent a package containing a summary of the guidelines (translated into Albanian) and the goals of the focus group.

Participants were identified through the same process as was used for the interviews. The focus groups were facilitated by experienced researchers (JM, SES) from Toronto, Canada, who also took field notes during the sessions. Two Albanian translators were in attendance. The focus groups were digitally recorded, and the recordings were transcribed and analyzed according to a process similar that used for the interviews. The researchers’ field notes were also analyzed thematically.

#### Phase four: in-person consensus meeting

We held an in-person meeting based on a nominal group process [23] to review and discuss the results of phases one to three. During the meeting, we presented the results of the survey, interviews, and focus groups, and asked attendees to discuss and rate the importance of each barrier to the implementation of the guidelines. We also asked them to rate the relevance and importance of each recommendation in the WHO guidelines. An electronic audience response system was used, whereby each participant voted and results of the vote immediately available for review. Consistent with the RAND appropriateness method [24], ratings were based on a nine-point Likert scale. The purpose of this ranking exercise was to identify the extent of consensus and to prompt reflection in preparation for further deliberation. Two Albanian translators were in attendance.

## Results

### Phase one: survey

**Figure 1:**
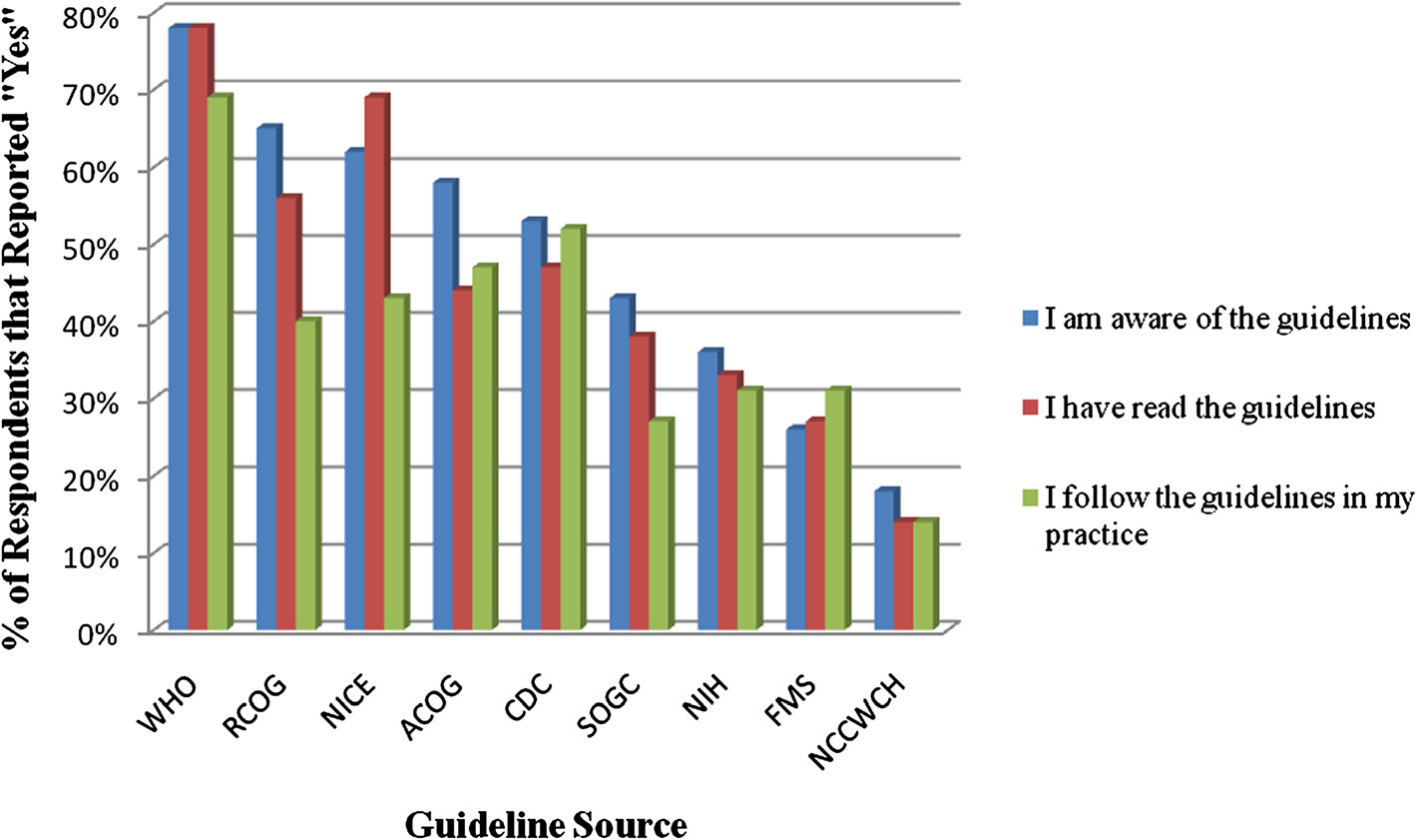
**Awareness and use of guidelines from various authorities.** WHO = World Health Organization (headquarters or regional office); RCOG = Royal College of Obstetricians and Gynaecologists (United Kingdom); NICE = National Institute for Health and Clinical Excellence (United Kingdom); ACOG = American Congress of Obstetricians and Gynecologists (USA); CDC = Centers for Disease Control and Prevention (USA); SOGC = Society of Obstetricians and Gynaecologists of Canada (Canada); NIH = National Institutes of Health (USA); FMS = Finnish Medical Society (Finland); NCCWCH = National Collaborating Center for Women’s and Children’s Health (USA).

A total of 39 responses to the survey were received. One-half (50%) of the respondents worked in a community setting, 22% in a hospital, and the remaining 28% in other settings (e.g.*,* University Clinical Center and non-governmental organizations). Participants consisted of family physicians and general practitioners (47%), obstetricians (22%), midwives (16%), nurses (6%), a pediatrician (3%), a biochemist (3%), and a medical student (3%). Participants had been in practice for an average of 11.6 years (range 1 to 31 years). Results of the survey are available in Table 1 and in Figure 1. Participants were most commonly aware of (78%) and had read (78%) the WHO guidelines but awareness did not always translate into use (67%) of these guidelines in practice. (Figure 1) Perceived barriers to implementation of the WHO postpartum hemorrhage guidelines included lack of access to appropriate equipment in their setting and lack of cooperation across colleagues within the healthcare system (Table 1).

**Table 1:** Responses to a survey about implementation of World Health Organization guidelines on postpartum hemorrhage

**Barriers**	**Mean**	**Standard deviation**
The equipment in my setting isn’t adequate for implementing the guidelines	2.83	1.15
Other clinicians in my setting won’t cooperate with implementing the guidelines	2.82	1.24
The facilities in my setting aren’t adequate for implementing the guidelines	2.58	1.30
I am isolated from knowledgeable colleagues with whom to discuss the guidelines	2.53	1.22
Other staff in my setting aren’t supportive of implementing the guidelines	2.50	0.92
The guidelines aren’t readily available in my setting	2.44	1.15
The skill set required to implement the guidelines is not available in my setting	2.37	1.07
The guidelines are not applicable to my setting	2.19	1.11
There is not a documented need to change practice according to the guidelines	2.16	0.96
I don’t have enough authority to change patient care procedures	2.11	1.02
The research in the guidelines hasn’t been replicated	2.06	1.00
I don’t feel capable of evaluating the quality of the guidelines	1.84	0.90
I feel that the benefits of changing practice will be minimal	1.84	1.17
There isn’t sufficient time on the job to implement new ideas	1.83	0.92
Administration in my setting won’t allow implementation of the guidelines	1.82	0.88
The research has methodological problems	1.82	1.01
I am unaware of any guidelines on these topics	1.79	1.13
The guidelines don’t provide guidance on implementation	1.75	1.00
I don’t see the value of implementing the guidelines in practice	1.74	1.10
I don’t have time to read guidelines	1.74	0.93
The guidelines report conflicting results	1.72	0.96
I don’t know whether to believe the guidelines	1.72	1.13
The guidelines aren’t readable	1.72	1.02
I don’t like trying new ideas	1.68	1.00
The recommendations in the guidelines aren’t justified	1.67	0.97
The implications for practice aren’t clear in the guidelines	1.67	0.84
The guideline recommendations aren’t reported clearly	1.67	0.91
The guidelines aren’t relevant to my practice	1.67	1.08
I don’t see the benefit of the guidelines	1.53	1.02

Response options: 1 = to no extent; 2 = to a small extent; 3 = to a moderate extent; 4 = to a great extent.

### Phase two: interviews

Of nine people invited to participate in telephone interviews, five agreed to do so; the small number of participants related to difficulty in recruiting participants who could speak English.

Themes identified during the interviews focused on barriers to guideline implementation. No comments were provided on facilitators to implementation, despite probing. No barriers were identified at the level of the patient, but several were noted at the level of the healthcare provider, including lack of awareness of the guidelines, lack of ability to access the guidelines (including lack of Internet access), lack of ability to monitor guideline implementation, lack of access to relevant medications (including oxytocin and magnesium sulphate), and lack of ability to smoothly and appropriately refer and transfer patients to large urban centers.

In addition, many barriers to implementation were identified at the level of the healthcare system, mostly attributed to the post-conflict situation. The war occurred in 1998 – 1999, and Kosovo declared its independence from Serbia in 2008, although this status is not universally recognized by other countries. Kosovo includes several Serbian-majority areas, and Serbia does not recognize Kosovo as an independent country. The conflict and its aftermath have significantly and adversely affected the infrastructure in Kosovo, including healthcare, and this situation was one of the most prominent themes revealed by the interviews. For example, since the conflict there has been no consistent mechanism for collecting healthcare data across the country. Similarly, respondents felt that healthcare has not been funded adequately in the post-conflict period and that this lack of funding has affected quality of care. For example, certain medications and equipment mentioned in the guidelines are not available to clinicians in Kosovo. There has been a growth in the development of privately insured healthcare, but the data from this insurance system are not publicly available and the quality of care provided under the system is unclear. The conflict also led to disintegration of healthcare across rural and urban centers, which has affected transitions in care and the communication between clinicians and policy makers. For example, participants emphasized fragmentation and lack of integration within the healthcare system since the conflict. Other challenges to implementation included high turnover of staff both within healthcare organizations and within the Ministry of Health, which has impaired clinicians’ ability to develop ongoing relationships with policy makers.

### Phase three: focus groups

A total of 19 people participated in the two focus groups. Participants’ demographic characteristics are presented in Table 2. Eighty-nine percent of participants were from Pristina. Participants included gynecologists (37%) and midwives/nurses (26%). Forty-two percent of participants worked in a hospital setting. Themes identified in the focus groups are presented in Table 3. The majority of the discussion focused on barriers to implementation. Quotations illustrating the themes are provided in italics below and have been translated into English as necessary.

**Table 2:** Demographic characteristics of participants in focus groups and consensus meeting

**Characteristic**	**No. (%) of participants (*n*** **= 19)**
Gender	
Male	8 (42)
Female	11 (58)
Age	
< 30	2 (11)
30 – 40	8 (42)
41 – 50	5 (26)
51 – 60	1 (5)
>60	
Location of practice	
Pristina	17 (89)
Other	2 (11)
Type of practice	
Community setting	1 (5)
Hospital-based setting	8 (42)
Other	10 (53)
Profession	
Gynecologist or obstetrician (practicing or teaching)	7 (37)
Nurse or midwife (practicing or teaching)	5 (26)
Employee of government organization	4 (21)
Employee of non-governmental organization	1 (5)
Other	2 (11)
Time in current profession (yr)	
< 2	1 (5)
2 – 5	10 (53)
6 – 10	7 (37)
>10	1 (5)

**Table 3:** Barriers identified in focus group and ratings from consensus meeting

**Barriers**	**Mean rating**	**Standard deviation**	**Range**
Lack of access to medications (e.g.*,* oxytocin).	9.00	0	(9 – 9)
Lack of integration of healthcare resources (e.g.*,* medications, equipment, healthcare personnel) across the system.	8.95	0.05	(8 – 9)
Lack of healthcare/clinical data (e.g.*,* for medication use, hospital diagnoses, complications).	8.81	0.15	(8 – 9)
Lack of ability to monitor implementation of guidelines.	8.76	0.18	(8 – 9)
Lack of ability to document and monitor current practice.	8.66	0.32	(7 – 9)
Lack of communication between professional associations and the Ministry of Health around guideline development.	8.63	0.23	(7 – 9)
Lack of integration of clinical care across the system.	8.60	0.34	(7 – 9)
Lack of accountability for guideline implementation.	8.57	0.35	(7 – 9)
Lack of communication between professional associations and the Ministry of Health around guideline dissemination and implementation.	8.43	0.35	(7 – 9)
Lack of agreement between professional associations and the Ministry of Health around data required for monitoring guideline implementation.	8.37	0.63	(6 – 9)
Lack of agreement with recommendations from guidelines.	8.32	2.42	(2 – 9)
Lack of communication across healthcare provider groups (e.g.*,* physicians, nurses, midwives).	8.26	2.89	(2 – 9)
Lack of process for identifying local opinion leaders/champions.	8.19	0.63	(7 – 9)
Lack of continuing education/continuing professional development on guideline use and implementation (capacity building).	8.10	0.89	(5 – 9)
Lack of a process for prioritising guidelines and recommendations for implementation.	8.09	0.56	(7 – 9)
Lack of undergraduate training (for medical and midwifery students) on guideline use and implementation.	7.85	0.93	(6 – 9)
Lack of clarity about who is responsible for implementing guidelines (e.g.*,* Ministry of Health and/or professional associations).	7.84	2.17	(5 – 9)
Lack of awareness of the protocols/recommendations from the guidelines.	7.70	5.31	(1 – 9)
Lack of awareness of the significance of the clinical problem.	7.21	3.43	(2 – 9)

Response scale: 1 = extremely unimportant; 5 = neither unimportant or important; 9 = extremely important.

### Barriers to implementation at healthcare provider level

Several barriers at the level of the healthcare provider were discussed. For example, some clinicians were not aware of the methods used to develop the WHO guidelines (‘he’s asking questions in terms of how the recommendations, how the guidelines were drafted’) and therefore did not find them ‘trustworthy’. Similarly, clinicians were concerned that the recommendations had been created without the involvement of relevant clinicians. Participants questioned whether recommendations that could not currently be implemented in Kosovar facilities (because of a lack of resources or equipment) should be included in the development of care pathways. For example, the WHO guidelines recommend a procedure that is currently not available in Kosovo, but several obstetricians suggested that it should be retained in the contextualized guidelines because it may become available as healthcare infrastructure improves. This issue relates to the ‘aspirational’ nature of guideline recommendations:

‘Sometimes you don’t, you cannot perform that, so I don’t see that like to exclude the embolization…. but I see that as an opportunity for you that you see that you have to do this and you don’t have conditions, and you have to ask for fulfilling this, in the training, in the equipment, in the materials.’

Other participants said that retaining such recommendations in local guidelines could be confusing for clinicians and might foster disengagement from implementation efforts if they feel the guidelines are not relevant to their practice.

Participants were very concerned about their lack of ability to document and monitor current practice:

‘We come to the problem of how to collect this information that whether the protocol is being implemented and what to do with those not implementing the protocol.’

Many participants stated that ‘without measurement, improvement in care cannot be done’.

Additionally, deliveries in Kosovo are handled by both obstetricians and midwives, and communication across these groups was perceived as a challenge and a limitation to guideline implementation: ‘communication between the professionals is, is, uh, lacking...’.

However, there was keen interest from participants to continue engaging with each other on educational initiatives to meet this challenge.

Capacity building was identified as a further challenge to guideline implementation. Participants noted a lack of undergraduate training (for midwives and medical students) and continuing education relating to guideline use and implementation. Strategies to incorporate these topics in undergraduate curricula were discussed, including integration throughout the training period:

‘I think that there is enough space to, to, uh, to, to introduce this into our regular teaching program, or curricular, regular curricular, or, uh, inside some, uh, trainings and, uh, continual professional developing of the staff, medical workers and co-workers.’

### Barriers to implementation at healthcare system level

Several barriers at the level of the healthcare system were identified, including lack of communication between clinicians and policy makers*.* The Ministry of Health recently established a quality improvement portfolio, including a Kosovo Guidelines Advisory Committee with a mandate to develop and/or adapt guidelines. Representatives of this committee attended the focus groups, but none of the clinicians at the consensus meeting was involved with or aware of the committee’s efforts. The focus groups and subsequent consensus meeting represented the first time that representatives of this committee were able to engage with relevant clinicians, and this opportunity stimulated substantial discussion on how to continue such engagement. These discussions also highlighted a lack of clarity about who should be responsible for developing and implementing guidelines, the Ministry of Health or professional associations (e.g.*,* the Kosovo Obstetrics and Gynecology Association):

‘The Ministry of Health should manage health policies at the Kosovo level … the professional associations should deal with the professional side, drafting, formulation of protocols and then monitoring of implementation for its own, its own field.’

Subsequently in the meeting, suggestions were made that the professional groups should work in partnership on implementation of guidelines, and a strategy for developing a small working group was discussed. However, participants in the focus groups also identified a lack of trust between clinicians and policy makers. As a result, the clinicians were not convinced by the methods outlined by the Guidelines Advisory Committee and expressed concerns about the motives and capacity of the Ministry of Health and the Kosovo Guidelines Advisory Committee.

All participants identified the need for access to data describing the quality of care, including diagnoses, use of medications, and adverse events. However, Kosovo has no national or regional system that can be used to document or audit care. As a result, the impact of guidelines cannot be adequately evaluated, and the effectiveness of implementation efforts may therefore be unknown:

‘That’s why there is a need to have a mechanism which measures, uh, the impact and then reports back on, on the outcomes, and then you get to the revision.’

Lack of consistent access to relevant medications and equipment was identified in the focus groups:

‘It would be better to say that there is, there is access, some access, but it’s not regular and it’s not quality access.’

For example, the WHO guidelines recommend use of oxytocin to prevent postpartum hemorrhage, and this drug is on the list of ‘essential medications’ in Kosovo. However, several clinicians mentioned that oxytocin is not consistently available in all hospitals, such as those in smaller, more rural cities: ‘then there are time ranges we don’t have, uh, not even oxytocin’. Representatives of the Ministry of Health agreed to prioritize this issue.

### Facilitators of implementation at healthcare system level

Several participants (both clinicians and policy makers) suggested that punitive measures could be initiated to facilitate implementation of guidelines. For example, it was suggested that:

‘Implementation can be best, uh, best, uh, required, or the best means is the punitive measures at least for the, for the initial, uh, for the initial peers so that the others, uh, follow suit.’

The use of punitive measures was explored in detail, as were legal mandates to implement guidelines. Other participants expressed concern about the potential to ‘game’ the system if punitive strategies were used.

### Phase four: consensus meeting

Eighteen participants attended the one-day, in-person meeting, all of whom had participated in a focus group the previous day. The consensus meeting was also attended by two representatives from the WHO office in Kosovo and two representatives from WHO headquarters in Geneva. The barriers rated highest by participants in the consensus meeting were lack of access to medications (e.g.*,* oxytocin), lack of integration of healthcare resources (e.g.*,* medications, equipment, healthcare personnel) across the system, lack of healthcare/clinical data (e.g.*,* for medication use, hospital diagnoses, complications), and lack of ability to monitor implementation of guidelines (Table 3).

## Discussion

In this study, we took a multiphase approach to understanding the barriers and facilitators to guideline implementation in an LMIC. We also attempted to use the barriers identified to enhance understanding of how guidelines should be adapted to local contexts to optimize their implementation.

Several important barriers to implementation were identified in this study. First, lack of communication between clinicians and ministry representatives was seen as leading to duplication of effort in creating or adapting guidelines. Second, there was a lack of communication across clinical groups that provide obstetrical care and a lack of integration across the healthcare system as a whole, including rural and urban centers. This fragmentation was thought to have directly resulted from the conflict. Third, the conflict substantially and adversely affected healthcare infrastructure in Kosovo, which has resulted in an inability to monitor quality of care across the country; a particular challenge has been in providing and monitoring care in Serbian-majority regions. Furthermore, the impact on infrastructure has affected the ability to access required medications consistently and to smoothly transfer patients from rural to urban centers.

Another issue raised during this project was the appropriateness of including guideline recommendations perceived to be ‘aspirational’. Kosovo experienced a dramatic event with the conflict of 1998 – 1999, but with investment in education and healthcare infrastructure, it may be able to quickly recover, allowing implementation of the aspirational recommendations in the not-distant future. However, including these recommendations in current guidelines may run the risk of disengaging clinicians, who may not feel that the guidelines are relevant to them and their existing practice. This perception is an area that warrants additional research and should be explored across different LMICs.

Some of the results from this study are similar to those reported by others, who have highlighted the importance of contextualization when implementing guidelines in LMICs [25,26]. However, several issues specific to Kosovo were identified that directly relate to the impact of recent conflict on healthcare infrastructure. Strategies to overcome these barriers will likely need to be individualized according to the Kosovo healthcare system and its resources. Nonetheless, it may be beneficial for LMICs to share information about assessing determinants to implementation and about their implementation strategies.

Our study had several limitations. First, we are unsure of the overall survey response rate because we do not know how many people accessed the websites where it was posted. However, we used the survey results to inform the development of the interviews and focus groups and found consistency in themes across these results. Second, the rate of participation in interviews was poor, largely because of language and technology difficulties. As a result of these challenges, we held two focus groups before the in-person consensus meeting to collect additional data. The material from the focus groups further enriched the data gathered during the interviews. Third, as mentioned above, because this was conducted in a single country, the results may not be generalizable to other LMICs as some identified factors are unique to a country that has experienced recent conflict. However, many of the other identified barriers to implementation such as lack of ability to monitor implementation and lack of communication across stakeholders are applicable to most clinical settings.

The study also had several strengths, including use of the knowledge to action framework [7] and the CAN-IMPLEMENT resource [13] as well as application of multiple research methods. These various methods provided rich, detailed information, allowing a deep understanding of the challenges associated with implementing the WHO guidelines, which target the Millennium Development Goals. This information will guide the next steps in implementing these guidelines. For example, a local working group consisting of clinicians and Ministry of Health representatives has been established to develop an implementation plan. Moreover, although some of the challenges in Kosovo may differ from those in other LMICs, the approaches used may strengthen implementation efforts in those other settings.

## Abbreviations

GRADE: Grades of Recommendation Assessment Development and Evaluation); GREAT: **G**uideline development **R**esearch priorities, **E**vidence synthesis, **A**pplicability of evidence, and **T**ransfer of knowledge; HRP: Special Program of Research Development and Research Training in Human Reproduction; KT: knowledge translation; LMIC: low- or middle-income country; WHO: World Health Organization.

## Competing interests

SES is an associated editor for Implementation Science but was not involved with any decisions regarding this manuscript.

## Authors’ contributions

SES conceived the study, completed the data collection and its analysis and drafted the manuscript. JM participated in data collection and analysis, and in drafting and revising the manuscript. CM participated in data collection and analysis. SU and AMG provided input into the study design and draft manuscript. All authors reviewed and approved the final manuscript.

## Supplementary Material

Additional file 1 **Survey.** Interview guide. Click here for file
