# Supplementary material for: Determinants of implementation of maternal health guidelines in Kosovo: mixed methods study
Source: Implement Sci. 2013 Sep 9;8:108. doi: 10.1186/1748-5908-8-108 (PMC3846581; doi:10.1186/1748-5908-8-108)
Supplement: Additional file 1 — Survey. Interview guide. [file 1748-5908-8-108-S1.doc]

**Appendix 1. Survey**

We need your help! Recognizing the challenges to improving the quality of care and decreasing the risk of adverse events in all healthcare systems has created interest in knowledge translation (KT). One of the most challenging KT problems is to lower the high rates of maternal and perinatal mortality and morbidity in many countries. We are trying to identify priorities for guideline implementation in order to meet this challenge. We would like to understand your needs and priorities for this implementation initiative as well as barriers and facilitators to implementation. Your answers to this brief survey will guide this initiative.

By completing this survey, you are providing informed consent to participate in this process. All of your responses will be anonymised and data will be kept confidential. Data will only be summarized in an aggregate fashion.

Participants who complete the survey will be entered into a draw for free access to the Cochrane Library for 6 months. If you are interested in entering this draw we ask you to complete and send the attached information card which provides your details.

If you have any questions about participating in this survey, please contact xx at xx.

Participant ID____

1. Location of practice:

Community setting

Hospital-based setting

Other (specify)_____

2. I am a:

Nurse

Midwife

Family physician/general practitioner

Obstetrician

Pediatrician

Other(specify)

3. I have been in practice for __ years

4. There are many different organisations that have developed guidelines relevant to optimising maternal care. For each of the organizations below, please state whether you’re aware of their guidelines on maternal health and whether you’ve read their guidelines.

a. Centers for Disease Control and Prevention (USA)

I am aware of the guidelines Yes No

I have read the guidelines Yes No

I follow the guidelines in my practice Yes No

b. American College of Obstetrics and Gynecologists (USA)

I am aware of the guidelines Yes No

I have read the guidelines Yes No

I follow the guidelines in my practice Yes No

c. Finnish Medical Society Duodecim (FINLAND)

I am aware of the guidelines Yes No

I have read the guidelines Yes No

I follow the guidelines in my practice Yes No

d. US National Collaborating Center for Women’s and Children’s Health (USA)

I am aware of the guidelines Yes No

I have read the guidelines Yes No

I follow the guidelines in my practice Yes No

e. US NIH Consensus Statements (USA)

I am aware of the guidelines Yes No

I have read the guidelines Yes No

I follow the guidelines in my practice Yes No

f. NICE (National Institute for Health and Clinical Excellence) (United Kingdom)

I am aware of the guidelines Yes No

I have read the guidelines Yes No

I follow the guidelines in my practice Yes No

g. Royal College of Obstetrics and Gynecology (United Kingdom)

I am aware of the guidelines Yes No

I have read the guidelines Yes No

I follow the guidelines in my practice Yes No

h. Society of Obstetricians and gynecologists of Canada (Canada)

I am aware of the guidelines Yes No

I have read the guidelines Yes No

I follow the guidelines in my practice Yes No

i. World Health Organization (Headquarters or regional office)

I am aware of the guidelines Yes No

I have read the guidelines Yes No

I follow the guidelines in my practice Yes No

j. Other (specify):

_________________

I am aware of the guidelines Yes No

I have read the guidelines Yes No

I follow the guidelines in my practice Yes No

2. The WHO has created several guidelines including those on optimizing maternal care. For each of these guidelines, please indicate how relevant it is in your own practice setting.

| Guideline | 1  Not at  all relevant | 2  Slightly relevant | 3  Somewhat relevant | 4  Neutral | 5  Moderately  relevant | 6  Very relevant | 7  Extremely relevant |
| --- | --- | --- | --- | --- | --- | --- | --- |
| a. Postpartum hemorrhage prevention  (2007) |  |  |  |  |  |  |  |
| b. Postpartum hemorrhage management (2009) |  |  |  |  |  |  |  |
| c. Managing complications of pregnancy and childbirth  (2000) |  |  |  |  |  |  |  |
| d. Pregnancy, childbirth, postpartum and newborn care –  A guide for essential practice (2006) |  |  |  |  |  |  |  |
| e. Effective perinatal care guidelines |  |  |  |  |  |  |  |

3. The WHO has developed several guidelines on optimizing maternal care including those on: postpartum hemorrhage prevention and management and labor induction as mentioned above. For each of these guidelines, please indicate how you would rate its importance for implementation in your own practice setting.

| Topic | 1  Not at  all important | 2  Slightly important | 3 Somewhat important | 4  Neutral | 5  Moderately important | 6  Very Important | 7  Extremely important |
| --- | --- | --- | --- | --- | --- | --- | --- |
| a. Postpartum hemorrhage prevention (2007) |  |  |  |  |  |  |  |
| b. Postpartum hemorrhage management (2009) |  |  |  |  |  |  |  |
| c. Managing Complications of Pregnancy and Childbirth (2000) |  |  |  |  |  |  |  |
| d. Pregnancy, childbirth, postpartum and newborn care - A guide for essential practice (2006) |  |  |  |  |  |  |  |
| e. Effective perinatal care guidelines |  |  |  |  |  |  |  |

4. **Barriers**

These next items are from the **Barriers scale** which identifies what are the most important barriers to implementing research in practice. As you complete these questions, consider what the barriers are to implementing evidence from clinical practice guidelines focused on optimising maternal and infant health in your own setting.

Please rate the extent to which you perceive each of these items is a barrier to the use of evidence from clinical practice guidelines focused on optimising maternal and infant health.

| Item | 1  To no extent | 2  To a little extent | 3  To a moderate extent | 4  To a great extent |
| --- | --- | --- | --- | --- |
| a. I am isolated from knowledgeable colleagues with whom to discuss the guidelines |  |  |  |  |
| b. There is not a documented need to change practice according to the guidelines |  |  |  |  |
| c. I don’t feel capable of evaluating the quality of the guidelines |  |  |  |  |
| d. I don’t see the benefit of the guidelines |  |  |  |  |
| e. I don’t see the value of implementing the guidelines in practice |  |  |  |  |
| f. I feel that the benefits of changing practice will be minimal |  |  |  |  |
| g. I am unaware of any guidelines on these topics |  |  |  |  |
| h. I don’t like trying new ideas |  |  |  |  |
| i. I don’t have time to read guidelines |  |  |  |  |
| j. The skill set required to implement the guidelines is not available in my setting |  |  |  |  |
| k. The facilities in my setting aren’t adequate for implementing the guidelines |  |  |  |  |
| l. The equipment in my setting isn’t adequate for implementing the guidelines |  |  |  |  |
| m. There isn’t sufficient time on the job to implement new ideas |  |  |  |  |
| n. Other staff in my setting aren’t supportive of implementing the guidelines |  |  |  |  |
| o. I don’t have enough authority to change patient care procedures |  |  |  |  |
| p. Other clinicians in my setting won’t cooperate with implementing the guidelines |  |  |  |  |
| q. The guidelines are not applicable to my setting |  |  |  |  |
| r. Administration in my setting won’t allow implementation of the guidelines |  |  |  |  |
| s. The research in the guidelines hasn’t been replicated |  |  |  |  |
| t. The guidelines report conflicting results |  |  |  |  |
| u. I don’t know whether to believe the guidelines |  |  |  |  |
| v. The research has methodological problems |  |  |  |  |
| w. The guidelines don’t provide guidance on implementation |  |  |  |  |
| x. The recommendations in the guidelines aren’t justified |  |  |  |  |
| y. The guidelines aren’t readily available in my setting |  |  |  |  |
| z. The implications for practice aren’t clear in the guidelines |  |  |  |  |
| aa. The guidelines aren’t readable |  |  |  |  |
| bb. The guideline recommendations aren’t reported clearly |  |  |  |  |
| cc. The guidelines aren’t relevant to my practice |  |  |  |  |

**Appendix 2. Interview guide.**

Firstly, I’d like to ask you some basic demographic information so that we may better understand the characteristics of our participants.

1. What is your age: a) under 30, b) 30 to 40, c) 41 to 50, d) 51 to 60, e) older than 61.
2. Where do you practice? (city)
3. What is your title/role description/length of time in this role?

Let’s now start with the interview, the first question I have for you is…

1. Based on the WHO guidelines presented in the Information Package, what do you think are some of the key messages relevant inKosovo? And Why?
2. Do you anticipate any barriers or challenges to implementing these guidelines in Kosovo? What are these specifically?E.g. institutional barriers, profession-specific barriers, educational barriers, logistical barriers, resistance from individuals or professional groups, etc.
3. Are there any facilitators that you think could aid in the implementation of these guidelines in Kosovo? Could you provide any examples?
4. Do you or have you used other guidelines in the past? If so what were some of the things you liked about the guidelines? E.g. Accessibility
5. We are hoping to interview other stakeholders, for example members of the public, healthcare managers, healthcare professionals, healthcare workers, and policymakers. Do you know anyone who would fit into one of those categories who also speaks English, who might be willing to participate in an interview?
6. I have no further questions. Do you have anything more you would like to bring up or ask before we finish the interview?

Thank you very much for participating. You will receive an invitation to attend a 1.5 day meeting discussing the key messages, barriers, and facilitators to implementing these guidelines in Kosovo. Attendance is voluntary. Please feel free to contact me if you have any questions. Thank you again, ____________.
